# Polysaccharides isolated from *Cordyceps Sinensis* contribute to the progression of NASH by modifying the gut microbiota in mice fed a high-fat diet

**DOI:** 10.1371/journal.pone.0232972

**Published:** 2020-06-08

**Authors:** Lei Chen, Liangyu Zhang, Wendong Wang, Wei Qiu, Lei Liu, Anhong Ning, Jing Cao, Min Huang, Mintao Zhong

**Affiliations:** 1 Department of Microbiology, College of Basic Medical Sciences, Dalian Medical University, Dalian, PR China; 2 First Affiliated Hospital of Dalian Medical University, Dalian, PR China; East Tennessee State University, UNITED STATES

## Abstract

Various dietary fibers are considered to prevent obesity by modulating the gut microbiota. *Cordyceps sinensis* polysaccharide (CSP) is a soluble dietary fiber known to have protective effects against obesity and related diseases, but whether these effects induce any side effects remains unknown. The function and safety of CSP were tested in high-fat diet (HFD)-feding C57BL/6J mice. The results revealed that even though CSP supplementation could prevent an increase in body weight, it aggravated liver fibrosis and steatosis as evidenced by increased inflammation, lipid metabolism markers, insulin resistance (IR) and alanine aminotransferase (ALT) in HFD-induced obesity. 16S rDNA gene sequencing was used to analyze the gut microbiota composition, and the relative abundance of the *Actinobacteria* phylum, including the *Olsenella* genus, was significantly higher in CSP-treated mice than in HFD-fed mice. CSP supplementation may increase the proportion of *Actinobacteria*, which can degrade CSP. The high level of *Actinobacteria* aggravated the disorder of the intestinal flora and contributed to the progression from obesity to nonalcoholic steatohepatitis (NASH) and related diseases.

## 1. Introduction

Obesity, which is characterized by metabolic disorder and chronic low-grade inflammation, has become a widespread public health problem [1, 2]. The excessive accumulation of fat and dyslipidemia can increase the risk of type 2 diabetes, nonalcoholic fatty liver disease (NAFLD) and even liver cancer [3, 4]. Multiple factors contribute to the development of obesity, including energy consumption, high fat intake and the microbiome.

Over the past few years, numerous studies have demonstrated that the gut microbiota plays an important role in diet-induced obesity and related diseases [5–7]. Recently, research on animal models has demonstrated that some specific intestinal microorganisms could prevent diet-induced obesity. Additionally, research investigating therapeutic approaches to modify the intestinal flora such as diet, probiotics, synthetic preparations, and fecal transplants is underway [8, 9]. Several probiotics have been used in clinical trials to decrease obesity and insulin resistance indexes and to regulate blood lipids in subjects and achieved good results [10, 11]. Additionally, dietary fiber can also increase intestinal flora diversity and physical barrier function (such as tight junction protein) [12, 13] and regulate liver metabolism [14].

*Cordyceps sinensis*, as a traditional Chinese medicine, has been used for thousands of years in some Asian countries. Some studies revealed that the polysaccharides from *C*. *sinensis* with a high molecular weight have a potential protective function against diet-induced obesity by changing the composition of the gut microbiota [15]. However, traditional medicine has a high risk of drug-induced liver injury (DILI), which is characterized by hepatocyte injury in China [16]. In addition, some researchers have noted that long-term supplementation with soluble dietary fiber in diet-induced obesity could cause cholestasis, liver inflammation and jaundice liver cancer (HCC) by increasing bacteria that ferment fiber and *Proteobacteria* in the intestine [17]. The liver is important lipid metabolism, and the gut microbiota can produce hazardous substances that are transported to the liver by the gut-liver axis, which could lead to low-grade inflammation and even cause NAFLD and nonalcoholic steatohepatitis (NASH) in diet-induced obesity [18–20].

These findings suggest that we need to be aware of the liver toxicity caused by traditional Chinese medicines and soluble dietary fiber used as antiobesity therapies. Therefore, the aim of this study was to determine whether *C*. *sinensis* polysaccharide (CSP) supplementation has side effects, such as NASH, on mice with diet-induced obesity. More importantly, we aimed to elucidate the links between changes in gut microbiota and the effects caused by CSP in HFD-fed mice.

## 2. Materials and methods

### 2.1 Polysaccharide preparation

Total extract from *C*. *sinensis* was purchased from Xi’an Ruilin Biotech Company. One hundred grams of extract powder was dissolved in 400 mL of distilled water, and protein was removed using the Sevag method 5 times [21]. The resulting solution was added to 6 times the volume of 95% ethanol and kept at 4°C for 12 h. After centrifugation at 5000 rpm for 10 min, the precipitate was washed with anhydrous ethanol, dissolved in distilled water, dialyzed with distilled water for 72 h at 4°C and freeze-dehydrated to obtain crude polysaccharides [22].

Then, the crude polysaccharides were separated as in a previous study [23]. Briefly, the crude polysaccharides were resuspended in distilled water and loaded into a DEAE-52 ion exchange column (Beijing Solarbio Bioscience Co., Ltd., China). Different concentrations of a NaCl-Tris-HCl buffer solution were used to elute ingredients at a flow rate of 1.0 mL/min. Then, 3 mL per tube was collected consecutively, and the concentrations of polysaccharides and proteins were measured by the phenol-sulfuric acid method and a BCA protein assay kit, respectively (Jiangsu KeyGEN Bioscience Co., Ltd., China).

The polysaccharide elution fraction with the highest polysaccharide concentration was loaded onto a Sephadex G-100 gel column (Beijing Solarbio Bioscience Co., Ltd., China) with a flow rate of 2.5 mL/min, and a Tris-HCl buffer solution was eluted at a rate of 2 mL/min.

The elution fraction with the highest polysaccharide concentration was collected, dialyzed against double distilled water for 72 h and freeze-dried for the next experiment. Furthermore, the molecular weight and monosaccharide composition were measured by high-performance gel-permeation chromatography (HPGPC) and high-performance liquid chromatography, respectively.

### 2.2 Animal experiments

Eight-week-old male C57BL/6J mice (20±1 g) were obtained from Dalian Medical University and housed in a specific pathogen-free environment with a 12 h/12 h light/dark cycle at 25±2°C and 50±5% relative humidity. Before starting the experiments, the mice were fed a normal diet for 1 week to acclimate. Three mice were housed in each cage, and the mice were rotated to difference cages multiple times to make the fecal microbiomes homogeneous. After 1 week, the mice were randomly divided into three groups (6 mice per group, 3 mice per cage). One group of mice was fed a normal chow diet (NCD, containing 13.8% kcal from fat, 3.65 kcal/g, Jiangsu Xietong Bioscience Co., Ltd., China) as healthy controls (NCD group), and one group was fed a high-fat diet (HFD, D12451, containing 45% kcal from fat, 4.05 kcal/g) as a model control (HFD group). The other group (HFD+CSP group) was maintained on the HFD and administered 300 mg/kg/d CSP by oral gavage. Body weight was recorded weekly. After 8 weeks of experimental treatment, fresh stool samples were collected in sterilized tubes and immediately frozen at -80°C for microbiota analysis. And the mice were anesthetized by inhaling ether. Blood samples were collected from the orbital plexus and centrifuged at 4°C (1000 × g, 15 min) to obtain the serum. Then, the mice were sacrificed by cervical dislocation. Liver, epididymal fat and colon samples were precisely dissected, weighed and frozen at -80°C.

### 2.3 Histological examination

Freshly isolated liver and epididymal fat tissues were fixed with 4% paraformaldehyde overnight. Then, the samples were dehydrated, embedded in paraffin and sectioned at 6 μm for hematoxylin and eosin (H&E) staining. Frozen liver sections (8 μm) were stained with Oil red O. All of these samples were photographed by using a light microscope (Tokyo Olympus Corporation, Japan).

### 2.4 Biochemical analysis

The concentrations of total triglyceride (TG), cholesterol (CHO), low density lipoprotein (LDL), high density lipoprotein (HDL) and alanine aminotransferase (ALT) in the serum were detected by using commercial kits (Nanjing Jiancheng Bioengineering Institute, China) based on the manufacturer’s instructions. The protein levels of serum insulin, lipopolysaccharide (LPS), MCP-1, TNF-α, and IL-6 were measured by using commercial ELISA kits (Shanghai Langdun Biotechnology Co., Ltd., China).

### 2.5 Glucose and insulin tolerance analyses

On the last day of the seventh week, after the mice were fasted for 12 h, an intraperitoneal glucose tolerance test (IPGTT) was performed by intraperitoneally injecting a dose of d-glucose (1.0 g/kg body weight). At 0, 15, 30, 60, and 120 min, blood samples were collected from the tail vein, and glucose levels were detected by a YUWELL Automatic Blood Glucose Meter (Jiangsu Yuyue Co., Ltd., China). During the last week of the experiment, an insulin tolerance test (ITT) was carried out in mice that were fasted for 6 h. After an intraperitoneal injection with 1 IU/kg body weight human insulin (Novolin R, Denmark), the blood glucose concentrations of the mice were tested at 0, 15, 30, 60, and 120 min.

### 2.6 Western blot analysis

Total proteins were extracted from colon sample in RIPA buffer supplemented with PMSF. After grinding and centrifugation, the supernatant was evaluated with a BCA protein assay kit, and distilled water was added to keep each sample at the same concentration. Protein samples were separated by a 6% SDS-PAGE gel and transferred onto PVDF transfer membranes. After blocking with 5% nonfat milk for 1 h at room temperature, the membranes were first incubated with primary antibodies against GAPDH (1:2000, Proteintech), Occludin (1:1000, Proteintech) and Zonula occludens-1 (ZO-1, 1:1000, Proteintech) at 4°C for 12 h and then with the secondary antibody for 1 h, as described previously. The intensities of protein bands were quantified with Image-Lab software, and the values were normalized to GAPDH.

### 2.7 RNA isolation and real-time PCR

The total RNA from liver and epididymal fat tissues was extracted by using TRIpure (Beijing, Bioteke Co., Ltd., China), and the cDNA was synthesized by 2×Power Taq PCR MasterMix (Beijing, Bioteke Co., Ltd., China). Then, Real-time PCR was carried out by an Exicycler96 instrument (BIONEER, Korea) with SYBR Green Supermix (Beijing Solarbio Bioscience Co., Ltd., China). Values were normalized to the control, β-actin, by using the 2^−△△CT^ method. All the primer sequences are listed in S1 Table.

### 2.8 Gut microbiota analysis

Fecal bacterial genomic DNA from 18 samples was extracted by an E.Z.N.A. ® Stool DNA Kit (Omega, Inc., USA) according to the manufacturer’s instructions. The V3-V4 area of the 16S rRNA gene from different samples was amplified with the primers 338F (5'-ACTCCTACGGGAGGCAGCAG-3') and 806R (5'-GGACTACH- VGGGTWTCTAAT-3'). The 5' ends of the primers were tagged with barcodes specific to each sample and universal sequencing primers. The PCR conditions used to amplify the prokaryotic 16S fragments consisted of an initial denaturation at 98°C for 30 seconds; 35 cycles of denaturation at 98°C for 10 seconds, annealing at 54°C/52°C for 30 seconds, and extension at 72°C for 45 seconds; and a final extension at 72°C for 10 min. After purification, the PCR products were sequenced on an Illumina MiSeq platform, provided by LC-Bio (Hangzhou, China), according to the manufacturer's recommendations. Paired-end reads were assigned to samples based on their unique barcode, truncated by removing the barcode and primer sequence and merged using FLASH. The quality filtering of the raw tags was performed under specific filtering conditions to obtain high-quality clean tags according to the fqtrim (V0.94). After filtering the chimeric sequences, the sequences were clustered into the same operational taxonomic units (OTUs) based on a ≥ 97% similarity by Vsearch (v2.3.4). Representative sequences were chosen for each OTU, and taxonomic data were then assigned to each representative sequence using the Ribosomal Database Project (RDP) classifier. The differences among the dominant species in different groups were identified and multiple sequence alignment were performed using MAFFT software (V 7.310) to study the phylogenetic relationships among different OTUs. OTU abundance information was normalized using a standard sequence number corresponding to the sample with the least sequences. The alpha diversity of our samples was calculated with QIIME (Version 1.8.0). Beta diversity was calculated by principal coordinate analysis PCoA and cluster analysis by QIIME software (Version 1.8.0). The biomarker species between groups was identified by linear discriminant analysis (LDA) effect size (LEfSe) analysis.

### 2.9 Statistical analysis

GraphPad Prism version 7.0 (GraphPad Software, USA) was used to evaluate the statistical significance among groups by ANOVA followed by Tukey’s multiple comparison tests. Data are presented as the mean ± SEM, and *P*<0.05 was considered statistically significant.

## 3. Results

### 3.1 Structure characterization of CSP

**Fig 1:**
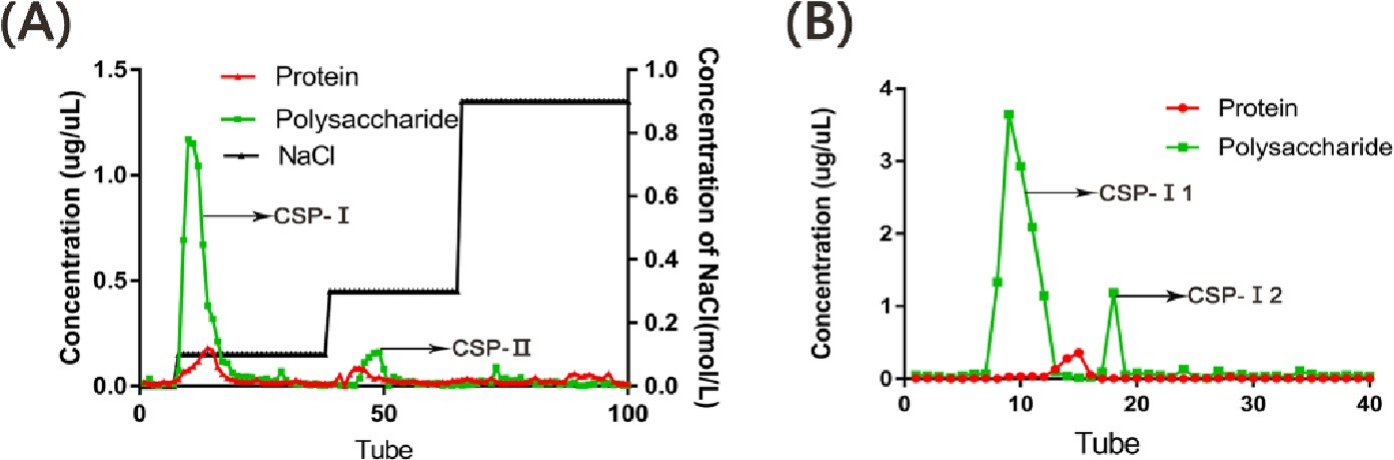
Polysaccharide was separated by Diethylaminoethyl Cellulose-52 (DEAE-52) and Sephadex G-100. A) The elution curve of polysaccharide fractions *Cordyceps sinensis* polysaccharide(CSP)- I and CSP-II on DEAE-52; B) The elution curve of polysaccharide fractions CSP-I 1 and CSP-I 2 on Sephadex G-100.

As shown in Fig 1A and 1B, the crude polysaccharides were separated into two fractions, CSP-I and CSP-II, by DEAE-52 chromatography. Then, G-100 chromatography was used to separate the higher fraction CSP-I and to remove proteins and ions. We obtained a single component polysaccharide CSP-I1 and named it CSP. The average molecular weight of CSP was 6.486×10^4^ Da, and the monosaccharide components of CSP are shown in Table 1. Based on the results, glucose was the main monosaccharide constituent of CSP.

**Table 1:** Monosaccharide compositions of CSP.

Monosaccharides	Glucose	Galaclose	Mannose	Arabinose	Rhamnose	Glucuronic Acid
Molar ratio of CSP	90262	526	317	264	184	151

### 3.2 Effects of CSP treatment on body weight, fat mass, liver weight and liver steatosis in HFD-induced obese mice

**Fig 2:**
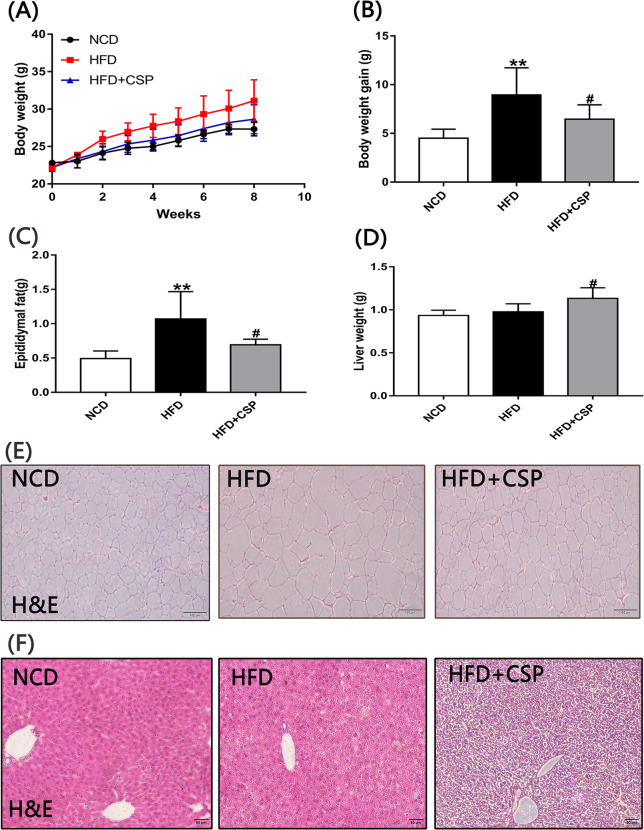
The effects of CSP on body weight, epididymal fat and liver. A) Time curve of body weight; B) Total body weight gain; C) Epididymal fat; D) Liver weight. Values are presented as mean±SE(n = 6). Differences were assessed by ANOVA and denoted as follows: *P<0.05; **P < .01 compared with NCD, *#P* < .05; *##P* < .01 compared with HFD, E and F) H&E of epididymal adipose tissue and liver tissue respectively.

As shown in Fig 2A, during the 8-week experiment, the body weight of the HFD group was higher than that of the HFD+CSP group at most time points. Compared with the HFD group (8.94±1.14 g), the HFD+CSP group showed significantly decreased body weight gain (6.45±0.60 g) (*p*<0.05) (Fig 2B). After comparing the weight of epididymal fat and the size of adipocytes (Fig 2C and 2E) in the HFD and HFD+CSP groups, we found that the weight of epididymal fat in the HFD+CSP group was remarkably lower than that in the HFD group (*P*<0.05), and the cell diameter of adipocytes was also decreased in the HFD+CSP group. However, the liver weight (Fig 2D) of the HFD+CSP group was significantly higher than that of the HFD group (*p*<0.05). Liver H&E staining (Fig 2F) revealed severe fibrosis, steatohepatitis changes and increased infiltration of inflammatory cells around the central vein of hepatocytes in the mice in the HFD+CSP group compared with the HFD group.

### 3.3 Effects of CSP on serum lipid and CHO levels, liver fat accumulation and liver function

**Fig 3:**
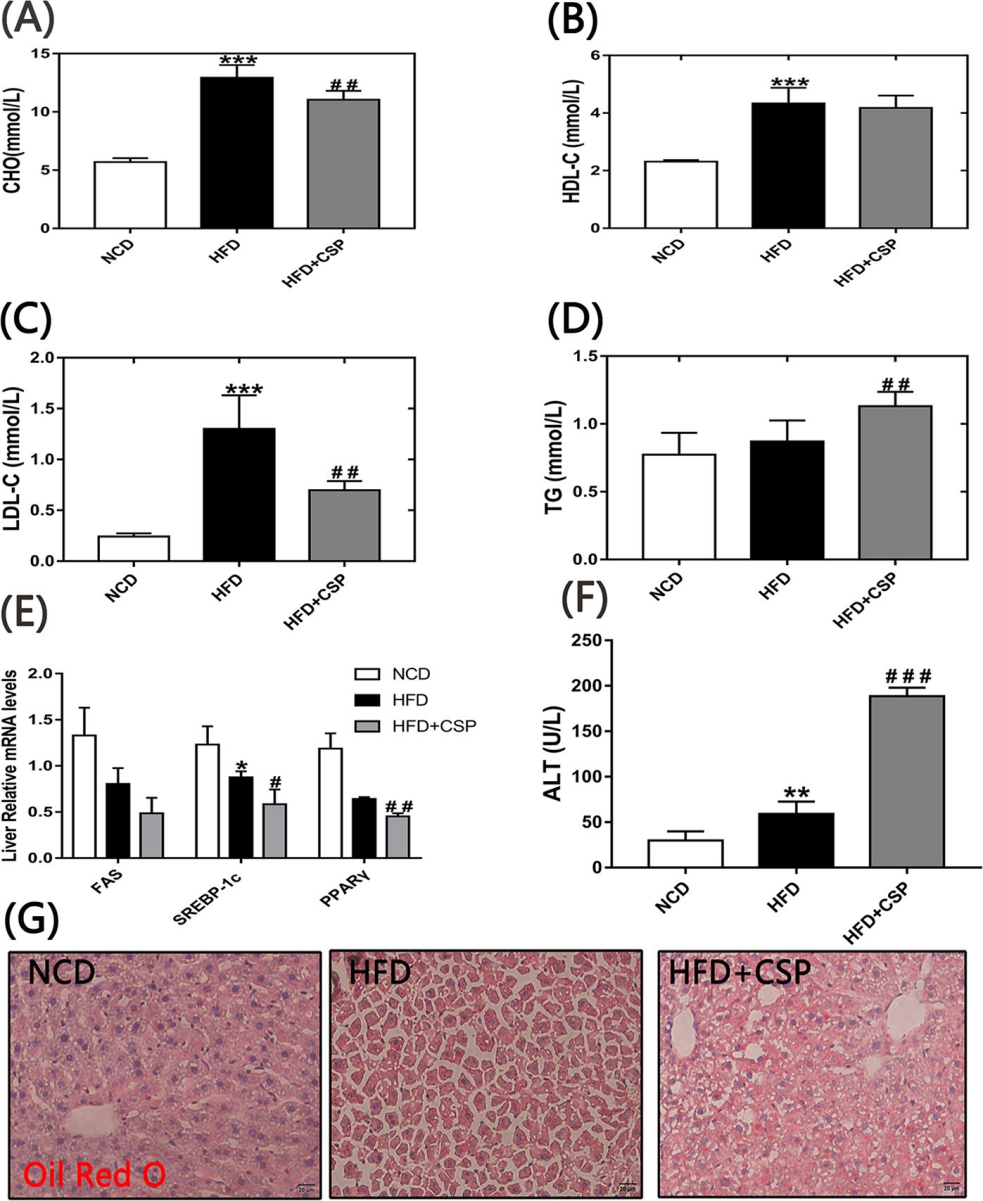
Effects of CSP on the serum lipids, expression of related genes and hepatic steatosis. A-D); Blood concentrations of CHO, HDL-C, LDL-C and TG respectively; E) Relative expression of *FAS*, *SREBP-1c* and *PPARy;* F) Blood concentration of ALT; G) Oil Red O staining of liver tissue sections. Differences were assessed by ANOVE and denoted as follows: *P<0.05;**P<0.01;***P<0.001 compared with NCD, *#P*<0.05; *##P*<0.01 compared with HFD.

After supplementation with CSP for 8 weeks, it was found that even though there was no significant difference in HDL-C (Fig 3A) between the HFD and HFD+CSP groups, the levels of LDL-C and CHO (Fig 3B and 3C) were notably decreased in the CSP-treated mice compared to the HFD-fed mice (*p*<0.01, *p*<0.01). Surprisingly, the TG concentration (Fig 3D) was remarkably increased in the HFD+CSP group compared with the HFD group (*p*<0.01). Next, we detected the expression of *FAS* and *SREBP-1c* genes, which control the de novo synthesis of endogenous fatty acids. As shown in Fig 3E, the expression levels of *SREBP-1c* and *FAS* in the HFD and HFD+CSP groups were both lower than those in the NCD group. The mRNA level of *SREBP-1c* in CSP-treated mice was significantly lower than that in HFD-fed mice. *PPARy* can inhibit the activation of hepatic stellate cells (HSCs), which are abnormally activated in NASH. We found that the mRNA levels of *PPARy* (Fig 3E) in the liver were dramatically decreased in the HFD+CSP group compared to the HFD group (*P*<0.01). In addition, the level of serum ALT (Fig 3F), which can represent liver function, was dramatically increased in the HFD+CSP group compared with the HFD group (*P*<0.001). From the Oil red O staining of the liver (Fig 3G), we found that the fat accumulation in the liver was higher in the HFD+CSP group than in the HFD group.

### 3.4 The effects of CSP on glucose tolerance and insulin resistance

**Fig 4:**
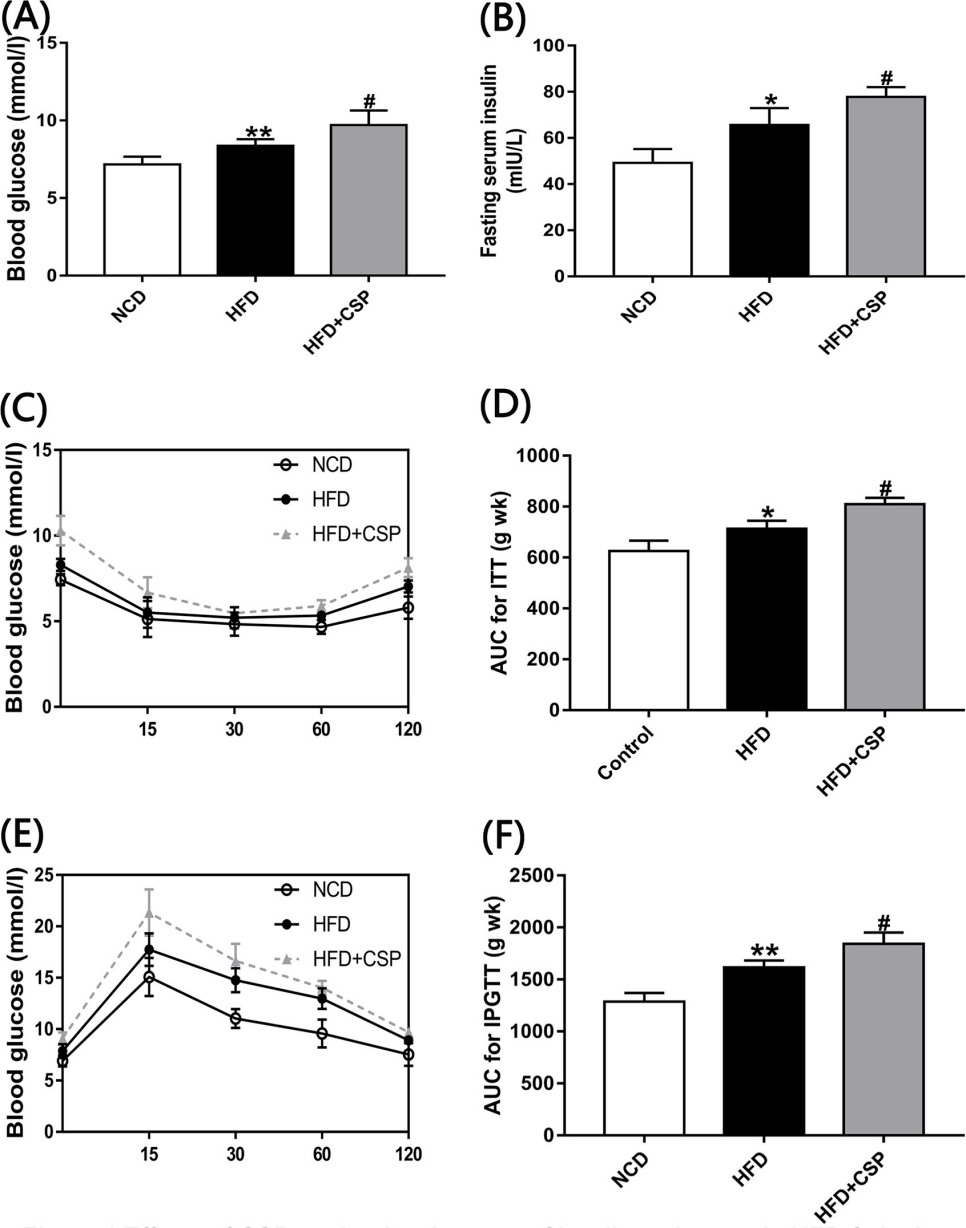
Effects of CSP on the development of insulin resistance in HFD-fed mice A) Fasting blood glucose; B) Fasting blood insulin; C) Insulin tolerance test(ITT); D) Area under the curve(AUC) For ITT; E) Intraperitoneal glucose tolerance(IPGTT); F) AUC for IPGTT. Differences were assessed by ANOVA and denoted as follows: *P<0.05; **P<0.01 compared with NCD, *#P*<0.05 compared with HFD.

As shown in Fig 4A and 4B, the levels of serum fasting glucose and insulin in CSP-treated mice were remarkably higher than those in HFD-fed mice (*P*<0.05). In addition, the ITT and IPGTT results (Fig 4C and 4E) showed that CSP could decrease insulin sensitivity. Furthermore, the calculated area under the curve (AUC) of the ITT and IPGTT (Fig 4D and 4F) in the HFD+CSP group was significantly larger than that in the HFD group (*P*<0.05), which could support the hypothesis that CSP can increase IR in HFD-induced obesity.

### 3.5 CSP increases inflammation in HFD-fed mice

**Fig 5:**
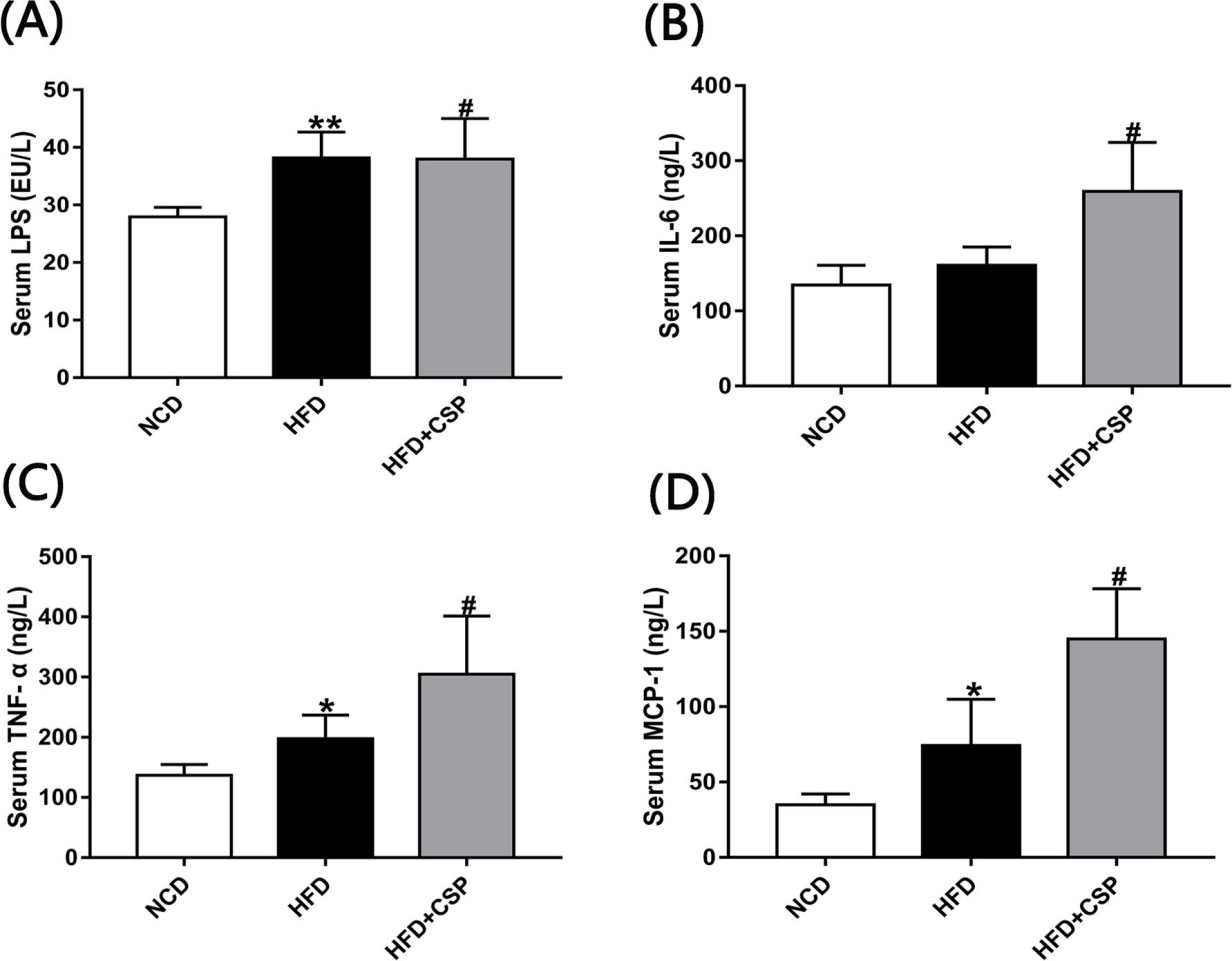
Effects of CSP on systemic inflammation in HFD-fed mice. A-D) Serum levels of LPS, IL-6 TNF-𝛂 and MCP-1 respectively. Difference were assessed by ANOVA and denoted as follows: *P<0.05; **P<0.01 compared with NCD, *#P<*0.05 compared with HFD.

Next, we investigated the level of LPS in different groups, which is known to play an important role in inflammation. Compared with the HFD group, CSP increased the concentration of serum LPS in HFD-fed obese mice (*p*<0.05) (Fig 5A). Then, we examined the inflammatory cytokine levels. As shown in Fig 5B, 5C and 5D, compared with the HFD group, the HFD+CSP group had dramatically increased serum TNF-α, IL-6 and MCP-1 levels (*p*<0.05). Moreover, in CSP-treated mice, the concentration of MCP-1, which is regarded as an indicator of NASH, was twice that in HFD-fed mice.

### 3.6 The effects of CSP on the protection of the gut intestinal barrier in HFD-fed mice

**Fig 6:**
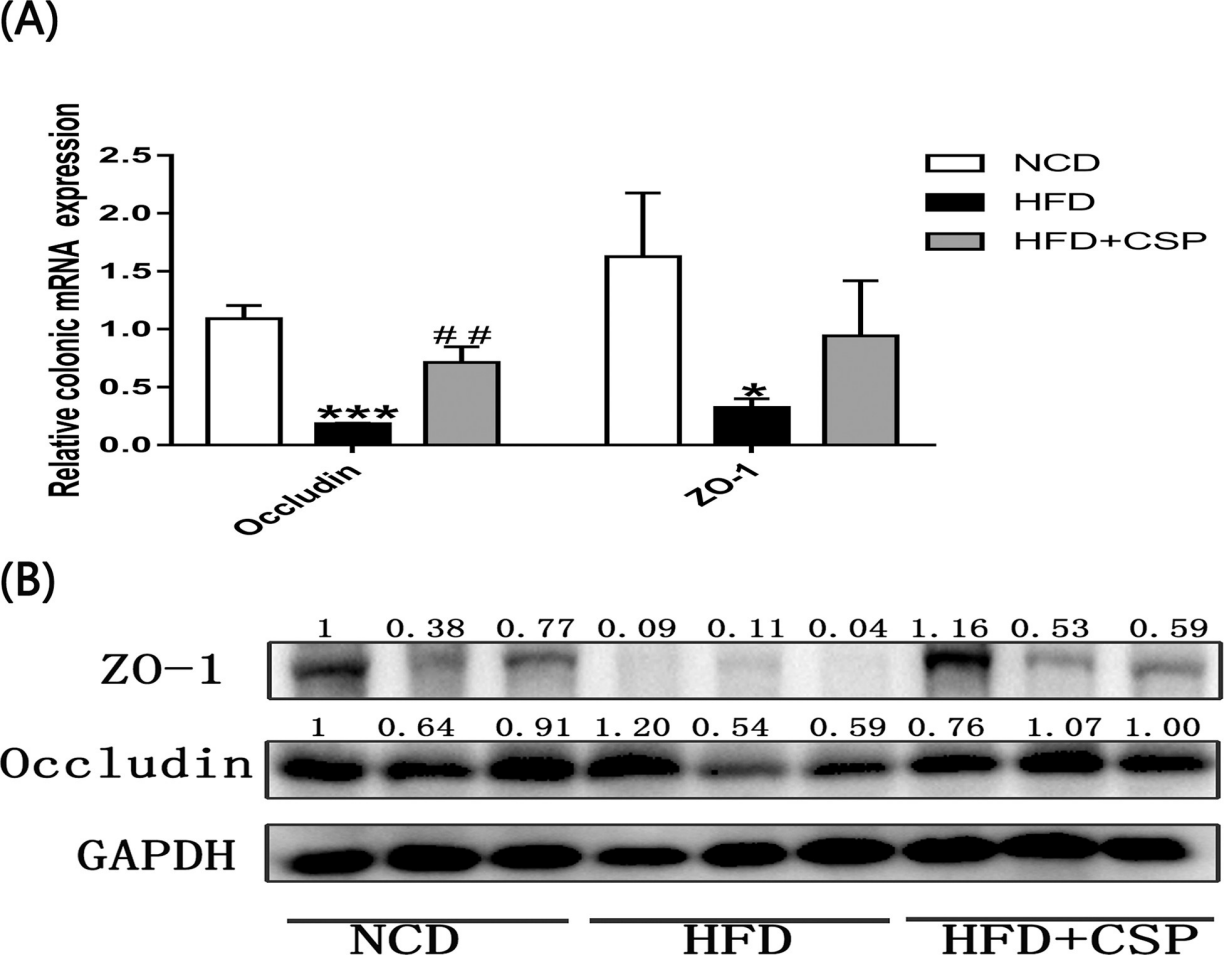
Effects of CSP on the relative expression of occludin and ZO-1. A) Relative expression of occludin and ZO-1 mRNA; B) Western blot analysis of occludin and ZO-1 proteins against GAPHD. Differences were assessed by ANOVA and denoted as follows: *P<0.05; ***P<0.001 compared with NCD, *##P*<0.05 compared with HFD.

We further measured the mRNA levels of *ZO-1* and *occludin*, which are considered to be important biomarkers in physical intestinal barrier function. As shown in Fig 6A, compared with HFD alone, CSP treatment notably elevated the mRNA concentration of *occludin* (*P*<0.01) and increased the mRNA level of *ZO-1*. In addition, the effects on the protein expression of *ZO-1* and *occludin* (Fig 6B) were consistent with the effects on the mRNA expression.

### 3.7 CSP alters the gut microbiota composition in HFD-induced mice

**Fig 7:**
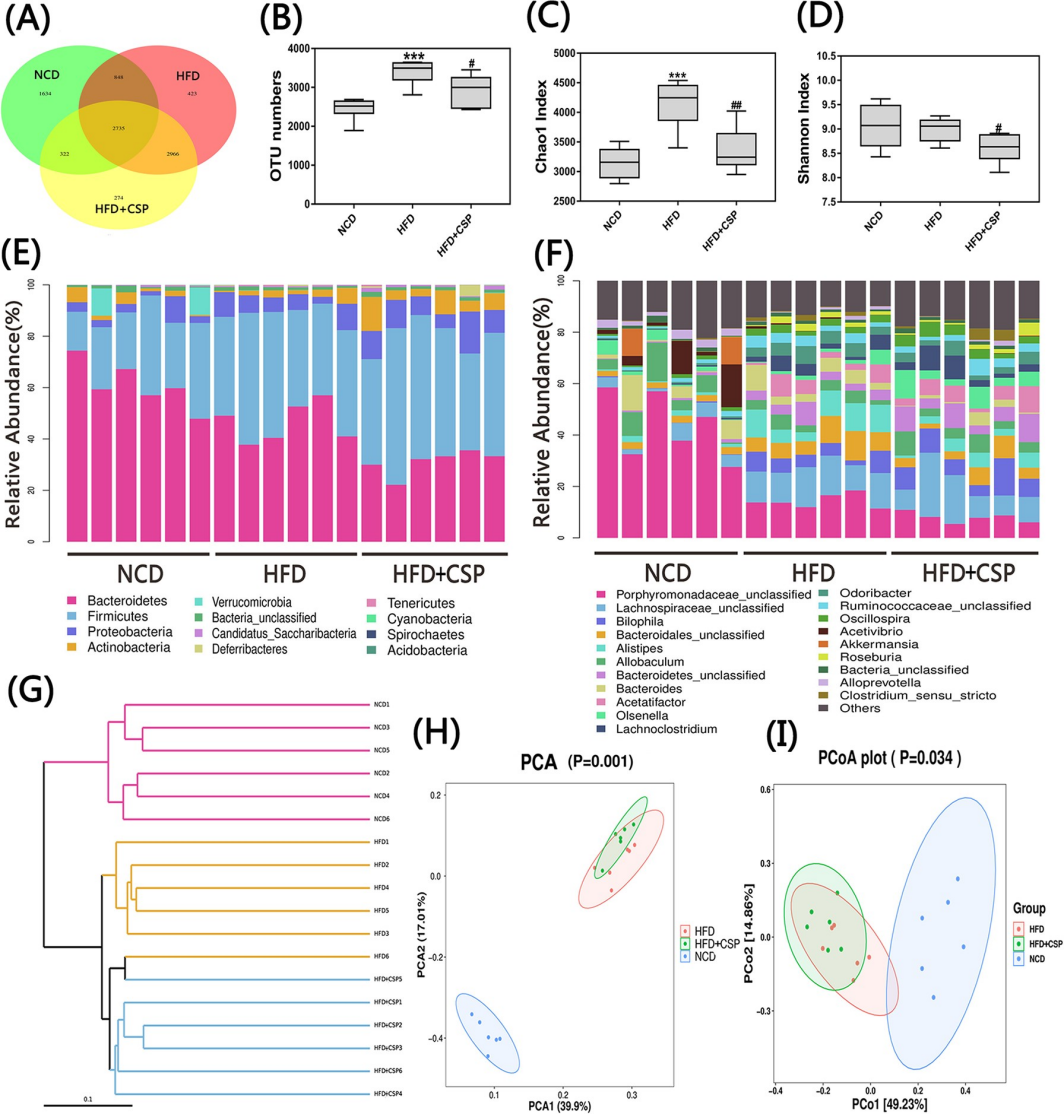
Effects of CSP on the gut microbiota structure. A) Venn diagram; B) OTU numbers; C) Chao1 index; D) Shannon index; E and F) Bacterial profile at phylum and genus level respectively. Values are presented as mean±SEM. (n = 6). Differences were assessed by ANOVA and denoted as follows: *P<0.05; **P<0.01; ***P<0.001 compared with NCD, *#P*<0.05; *#P*<0.01 compared with HFD. G) Unweighted UPGMA of all samples.;H) Plots of PCA; I) Plots of Weighted Unifrac-based PCoA.

To investigate the effects of CSP on gut microbiota composition, we sequenced the V3+V4 region of 16S rDNA genes in stool samples with Illumina MiSeq sequencing. A total of 364539 raw sequences were detected in 18 samples, and based on a 97% similarity level, all reads were clustered into OTUs. As shown in Fig 7A and 7B, Venn diagram analysis was performed to understand the richness in different groups, and the number of OTUs in the HFD-CSP group was significantly decreased compared with that in the HFD group (*p*<0.05). Next, we found that supplementation with CSP remarkably decreased the α-diversity of the HFD group compared with that of the HFD group according to the Chao1 index and Shannon index (Fig 7C and 7D; *p*<0.01 and *p*<0.05, respectively). At the phylum level, compared with HFD alone, CSP treatment not only decreased the relative abundance of *Bacteroidetes* but also increased the richness of *Proteobacteria* and *Actinobacteria* (Fig 7E). The changes at the genus level are shown in Fig 7F. In addition, the effects of CSP on the intestinal microbiota structure were revealed by an unweighted UniFrac cluster tree (Fig 7G), which was based on UPGMA. Furthermore, the structure of the gut microbiota among groups was also detected by using principal component analysis (PCA) and UniFrac distance-based PCoA. As shown in Fig 7H and 7I, these three groups presented significantly distinct microbiota profiles.

**Fig 8:**
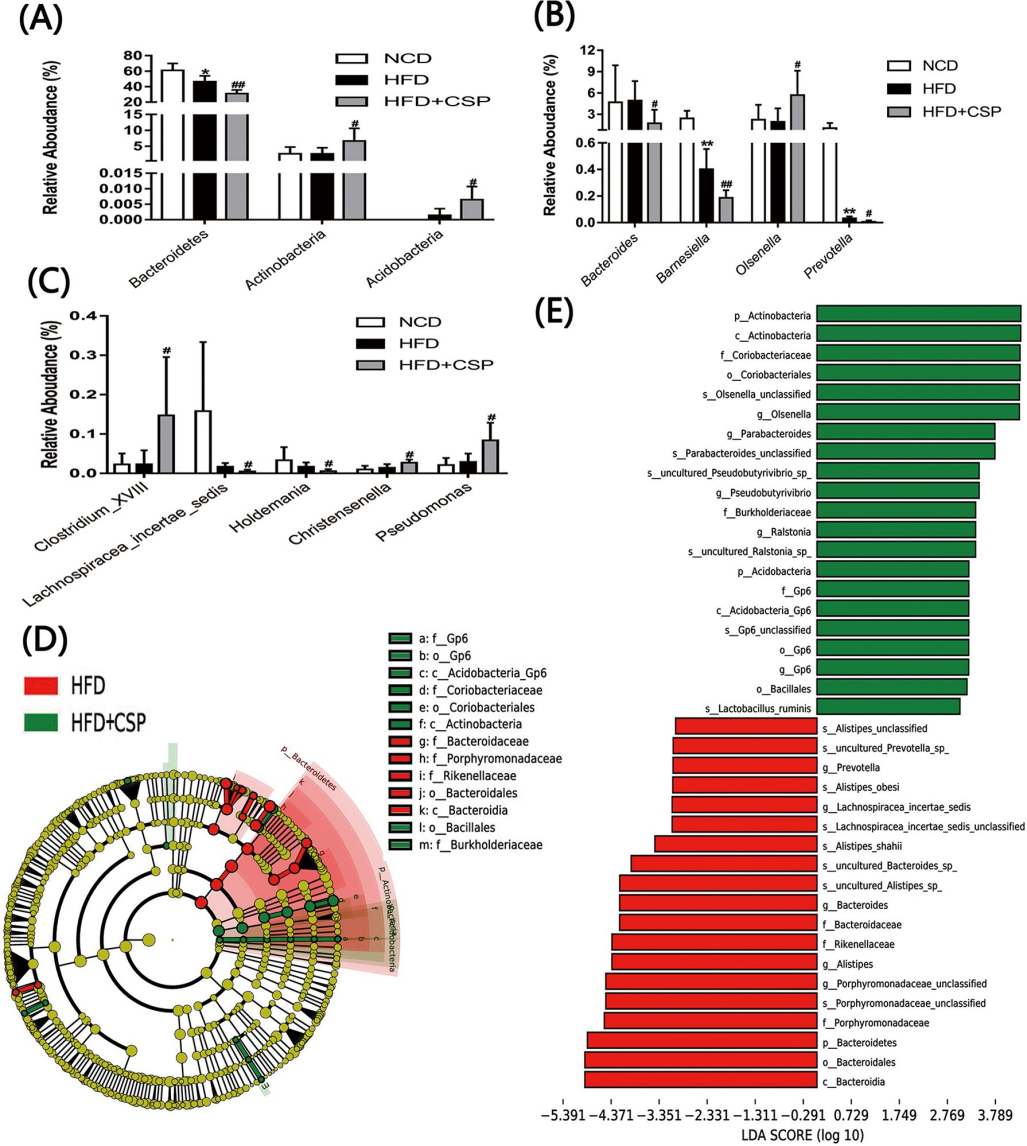
The effects of CSP on the gut microbiota composition and LEfSe analysis results. A) The relative abundance of gut microbiota at phylum level; B and C) The relative abundance of gut microbiota at genus level. Differences were assessed by ANOVA and denoted as follows: *P<0.05; **P<0.01 compared with NCD, *#P*<0.05; *##P*<0.01 compared with HFD. D and E) Cladogram showing the phylogenetic relationships of bacteria taxa and LDA scores between the HFD and the HFD+CSP group.

The specific changes in microbial composition in the three groups at various taxonomic levels are shown in Fig 8A, 8B, and 8C. We found that CSP dramatically decreased the relative abundance of *Bacteroidetes* but increased *Actinobacteria* and *Acidobacteria* at the phylum level in HFD-fed mice (*p*<0.01, *p*<0.05, *p*<0.05, respectively). To further investigate the differences between the HFD group and the HFD+CSP group, we compared the gut microbiota of these two groups at the genus level. As shown in Fig 8B and 8C, CSP treatment generated remarkable changes at the genus levels (5 genera were decreased, and 4 genera were increased).

Finally, the LEfSe method was used to identify high-dimensional biomarkers of the intestinal microbiota in the HFD and HFD+CSP groups. A comparison of the HFD+CSP group and the HFD group demonstrated that 21 phylotypes were increased, and 19 phylotypes were decreased (Fig 8E). As shown in Fig 8D, microbiota in the *Bacteroidetes* phylum, *Bacteroidia* class, *Bacteroidales* order, *Bacteroidaceae* family, *Rikenellaceae* family and *Porphyromonadaceae* family were enriched in the HFD-fed mice. However, the abundance of the *Actinobacteria* phylum, *Actinobacteria* class, *Coriobacteriales* order and *Coriobacteriaceae* family as well as the *Acidobacteria* phylum, *Acidobacteria_GP6* class, *GP6* order and *GP6* family were increased following CSP supplementation. The above results are also shown in the bar graph (Fig 8E).

## 4. Discussion

Although a large number of studies have reported that polysaccharides obtained from plants, fungi or animals, as a source of dietary fiber, have potential antiobesity functions by modulating the gut microbiota [24–27], the side effects of the soluble dietary polysaccharide extracted from *C*. *sinensis*, which is a traditional Chinese medicine, have not yet been reported. In this study, we purified polysaccharides from *C*. *sinensis* extract power and evaluated the monosaccharide composition and the molecular weight of the main polysaccharide. We found that the average molecular weight was 6.486×10^4^ Da and that it was mainly composed of glucose.

Dietary supplementation with CSP for 8 weeks significantly inhibited the increase in body weight gain in HFD-fed mice. In addition, it was also observed that the accumulation of epididymal fat and the cell diameter of adipocytes in mice in the HFD+CSP group were lower than those in mice in the HFD group (Fig 2C and 2E). These findings illustrate that CSP exerts clear protective effects on diet-induced obese mice, but the reason is still unknown. Next, we were surprised to find that the liver of CSP-treated mice was remarkably larger than that of HFD-induced mice (Fig 2D). An abnormal increase in liver weight is often accompanied by a change in liver structure and impairment of liver function [28–30], and NASH is the most common liver-related disease in diet-induced obesity [31]. Histopathological analysis and Oil red O staining of the liver in the CSP treatment group showed severe fibrosis, steatosis and inflammatory cell infiltration, which are characteristics of NASH [32, 33], but slight steatosis was observed in HFD-fed mice (Figs 2F and 3G).

As the liver controls blood lipid metabolism, we detected related indicators and found that the levels of CHO and LDL-C were decreased in the HFD+CSP group compared with the HFD group, and these CHO-related indices were consistent with some studies on the effect of prebiotics on antiobesity and related diseases [34, 35]. As a result of long-term HFD intake, LDL receptor activity was inhibited in the liver, so the concentrations of LDL-C in the HFD+CSP group and HFD group were both higher than those in the NCD group (Fig 3A and 3C). However, there were no significant differences in the level of serum HDL-C, which indicates a difference in reverse cholesterol transport (RCT) between the HFD+CSP group and the HFD group (Fig 3B). These results suggested that CSP has a potential antiobesity effect by inhibiting endogenous CHO without changing the capacity of RCT. Surprisingly, we found that the TG concentration of CSP-treated mice was notably higher than that of HFD-fed mice (Fig 3D). Accumulating evidence has shown that an abnormally high level of TG is characteristic of NASH [36, 37]. Furthermore, we checked the expression of genes related to the de novo synthesis of endogenous fatty acids, including Srebp-1c and FAS [38–40]. Although it had been reported that the expression of Srebp1-c and FAS were increased in HFD-fed mice [2, 41], some studies showed the opposite result [42, 43]. Our results were consistent with the later findings. The mRNA levels of both genes were decreased in CSP-treated mice compared to HFD-fed mice (Fig 3E). The consumption of a HFD will increase the levels of acyl-coenzyme A (acyl-CoA, the activated form of fatty acids), which is an allosteric inhibitor in the cells, and the high concentration of acyl-CoA will inhibit the activity of acetyl CoA carboxylase (ACC), a rate-limiting enzyme that catalyzes the first step of the fatty acid synthesis pathway that produces malonyl-CoA. Palmitic acid biosynthesis is controlled by the *SREBP-1c* and *FAS* genes. The low expression of *SREBP-1c* and *FAS* in the HFD+CSP group may suggest that the concentration of fatty acids in the hepatocytes of CSP-treated mice was high.

In addition, we found that compared with HFD alone, CSP treatment downregulated *PPARγ* in the liver (Fig 3E). Previous studies have shown that *PPARγ* can inhibit activation of HSCs and is present at a low level in the liver in NASH [44–46]. The above results suggest that CSP supplementation may contribute to the progression from obesity to NASH. To verify our hypothesis, we tested serum ALT and found that the concentration of ALT was significantly increased in CSP-treated mice compared with HFD-fed mice (Fig 3F). The ALT level was used to identify steatohepatitis and to predict NASH or moderate to severe advanced fibrosis in previous studies [47, 48]. The abnormal increase in ALT levels in the HFD+CSP group showed that CSP treatment induced severe NASH in HFD-fed mice. In addition, we observed that the concentrations of serum fasting glucose and insulin and the AUC for the ITT and IPGTT were increased in CSP-treated mice compared to HFD-fed mice (Fig 4A–4F). These findings suggest that CSP treatment increases the degree of IR in HFD-fed mice. IR often occurs in type 2 diabetes with weight loss. The slow increase in body weight in CSP-treated mice may suggest that CSP not only leads to NASH but also increases the risk of type 2 diabetes. In addition, IR-induced TG aggregation in hepatocytes is considered to be an important cause of NASH [49]. IR can increase the decomposition of peripheral fat and the release of free fatty acids (FFAs) in the blood. For this reason, the weight of epididymal fat and the cell diameter of adipocytes were decreased in CSP-treated mice. An increase in fatty acid intake by hepatocytes can lead to increased cytotoxicity via mitochondrial swelling, hepatocyte permeability, hepatocyte degeneration, necrosis and aggravated neutrophil infiltration [49].

Several reports also suggested that low-grade inflammation is a cause of IR in HFD-fed mice [50, 51]. In our study, serum biomarkers of inflammation were significantly increased in CSP-treated mice compared to HFD-fed mice (Fig 5B–5D). In particular, MCP-1, which is released from hepatocytes and activated HSCs, can promote the progression of NASH [52]. In addition, the increase in serum LPS, which is known to be mainly produced by gram-negative bacteria, suggested dysfunctional gut barrier function [53, 54]. Although many studies have shown a negative correlation between serum LPS levels and colonic intestinal barrier function [55, 56], our results revealed that CSP can reverse the reduction in colonic physical barrier markers caused by HFD. This is a controversial discovery. The increase in serum LPS content in the HFD+CSP group may be due to the transfer of harmful bacteria from the colon to the small intestine, resulting in the excessive proliferation of physical barrier cells in the small intestine and the absorption of LPS and chylomicrons (CMs) by the small intestinal epithelial cells [57, 58]. In addition to the intestinal physical barrier, the intestinal tract also includes an immune barrier, a chemical barrier and a biological barrier, which are associated with the composition of microorganisms in the intestinal tract [59, 60]. Damage to the biological barrier in the colon can also cause a bacterial product shift. Nevertheless, a previous study also suggested that intestinal barrier dysfunction can promote NASH; only a small proportion of patients have intestinal permeability and increased bacterial product shift [61]. This result indicates that CSP contributes to the progression of NASH in HFD-fed mice, which may be due to the excessive production of metabolites in CSP-treated mice that can easily diffuse across colonic barriers.

Numerous studies have suggested that dietary fiber can improve the intestinal barrier and inhibit inflammation via short-chain fatty acids (SCFAs) digested by gut microbiota and that the intake of dietary fiber can prevent some diseases and contribute to health [62–64]. Processed foods from purified fiber can have terrible consequences in some cases. For example, researchers found that mice fed inulin-enriched food developed marked colitis when exposed to DSS [65]. These findings suggest that purified dietary fiber may have similar side effects on intestinal flora regulation. Additionally, changes in the gut microbiota may contribute to the progression of NASH. Therefore, we evaluated the intestinal microbiota through 16S sequencing and found that the relative abundance of *Bacteroidetes* phylum was remarkably decreased in the HFD+CSP group compared to the HFD group (Fig 8A). *Bacteroidetes* are generally regarded as the main decomposers of dietary fiber and are also negatively correlated with NASH [66, 67]. In addition, CSP increased the proportion of the *Actinobacteria* phylum, which was mainly caused by increasing the level of *Olsenella* bacteria. As in a previous study, the microbiota of piglets in a dietary fiber group contained increased *Actinomycetes* bacteria, which promoted fiber degradation and butyric acid production [68]. This means that CSP can be degraded by *Actinobacteria* bacteria, resulting in butyric acid production. *Actinobacteria* was notably increased in NAFLD patients, and the degree of fatty liver and abnormal liver function were negatively correlated with the abundance of the *Actinobacteria* phylum and *Proteobacteria* phylum [67, 69]. Therefore, the increase in the relative abundance of the *Actinobacteria* phylum induced by CSP administration may contribute to the progression from the mild NAFLD induced by HFD to NASH. Zheng et al. also reported that the *Actinomycetes* phylum was increased in early HCC patients [70]. Moreover, the abundance of the *Acidobacteria* phylum was increased in the HFD+CSP group compared to the HFD group (Fig 8A). You et al. noted augmentation of the *Acidobacteria* phylum in severe fatty liver hepatitis mice induced by HFD and antibiotics [71].

At the genus level, the CSP-treated mice showed a significant decrease in the *Barnesiella* genus (Fig 8B), which has anti-inflammatory properties, produces SCFAs and is positively associated with abnormal lipid metabolism [72]. Additionally, a reduction in the *Prevotellaceae* genus and the *Lachnospiraceae incertae sedis* genus (Fig 8B and 8C), which were also found to be significantly reduced in maternal HFD-induced changes in 3-week-old offspring mice, was also observed. These bacteria have the ability to ferment carbohydrates and produce SCFAs, and the abundance of these genera was negatively correlated with obesity, diabetes, cardiovascular disease and metabolic syndrome [73–75]. The level of *Lachnospiraceae incertae sedis* was also significantly reduced in colorectal cancer rats compared with normal rats [75]. Surprisingly, *Holdemania*, a potentially harmful bacteria [76], was observed at a low level in the CSP-treated mice (Fig 8C). We found that the relative abundance of *Christensenella* was increased by nearly threefold due to the administration of CSP. Mie et al. found that *Christensenella* was associated with high fasting blood glucose concentrations [77]. The proportion of the *Clostridium_XVIII* genus, which mainly contains pathogenic species, was notably increased in CSP-treated mice compared to HFD-fed mice (Fig 8C) and was positively correlated with serum and hepatic lipid profiles [78]. Furthermore, CSP consumption increased the *Olsenella* genus (Fig 8C). In at study on the effect of pectin on NAFLD prevention in mice, improvements in NAFLD were associated with a decrease in *Olsenella*, which can form acetic acid and propionic acid [79]. It was also found that bacteria in the *Pseudomonas* genus, which can produce LPS, was also enriched in the HFD+CSP group (Fig 8C). Choi et al. suggested that an increase in *Pseudomonas panacis* (phylum, *Proteobacteria*) can disrupt the insulin signaling pathway in adipose tissue by the production of bacteria-derived extracellular vesicles (EVs) [80]. A seminal study also revealed the remarkable correlation of 3-(4 hydroxyphenyl) lactate, which is a gut microbiota metabolite, with liver fibrosis [81]. Treatment with phenylacetic acid, a novel microbial metabolite, led to TG accumulation in primary human hepatocytes and increased TG content in the liver in mice [82]. These findings also suggest that CSP contributes to the progression of NASH in HFD-fed mice and that the mechanism may involve some microbial metabolites produced by bacteria with high abundance in CSP-treated mice.

## 5. Conclusion

CSP treatment reduced body weight and epididymal fat in HFD-fed mice but had side effects on the liver. Furthermore, supplementation with CSP increased the levels of inflammation, serum TG, blood glucose, insulin, and ALT and the degree of liver fat accumulation, fibrosis, hepatic steatosis and steatohepatitis. CSP was mainly degraded by *Actinobacteria*, and the high abundance of *Actinobacteria* aggravated the disorder of the intestinal flora and contributed to the progression from obesity to NASH and related diseases. However, the changes in intestinal flora and corresponding metabolites require further investigation. Overall, these findings provide new insights suggesting that purified fibers, especially fermentable fibers from traditional Chinese medicine or others, should be used cautiously as approaches to improve metabolic diseases as these molecules alter the gut microbiota.

## Supporting information

S1 Table Primers used for quantitative real-time PCR analysis. (DOC)

S1 Raw images (TIF)
