# Supplementary material for: Polysaccharides isolated from Cordyceps Sinensis contribute to the progression of NASH by modifying the gut microbiota in mice fed a high-fat diet
Source: PLoS One. 2020 Jun 8;15(6):e0232972. doi: 10.1371/journal.pone.0232972 (PMC7279895; doi:10.1371/journal.pone.0232972)
Supplement: S1 Table — (DOC) [file pone.0232972.s001.doc]

**Table 1. Primers used for quantitative real-time PCR analysis.**

| **Primer name** | **Sequences of primers** | **Tm (°C)** |
| --- | --- | --- |
| *FAS* | Forward:5'-AACCAGACTTCTACTGCGATTC  Reverse: 5'-TCAACAACCATAGGCGATTT | 55.7 |
| *Srebp-1C* | Forward:5'-CAGAGCCGTGGTGAGAAGC  Reverse: 5'-GCAAGAAGCGGATGTAGTCG | 58.7 |
| *PPARγ* | Forward:5'-TAGAACCTGCATCTCCACC  Reverse: 5'-CACAGACTCGGCACTCAAT | 53.3 |
| *Occludin* | Forward:5'-TTTCCTTAGGCGACAGCG  Reverse: 5'-CCATCTTTCTTCGGGTTT | 57.1 |
| *ZO-1* | Forward:5'-TGCCTCGAACCTCTACTC  Reverse: 5'-GTGGTGGAACTTGCTCAT | 50.0 |
| *β-actin* | Forward:5'-CTGTGCCCATCTACGAGGGCTAT  Reverse: 5'-TTTGATGTCACGCACGATTTCC | 64.5 |
