# Supplementary figures and images for: Polysaccharides isolated from Cordyceps Sinensis contribute to the progression of NASH by modifying the gut microbiota in mice fed a high-fat diet
Source: PLoS One. 2020 Jun 8;15(6):e0232972. doi: 10.1371/journal.pone.0232972 (PMC7279895; doi:10.1371/journal.pone.0232972)

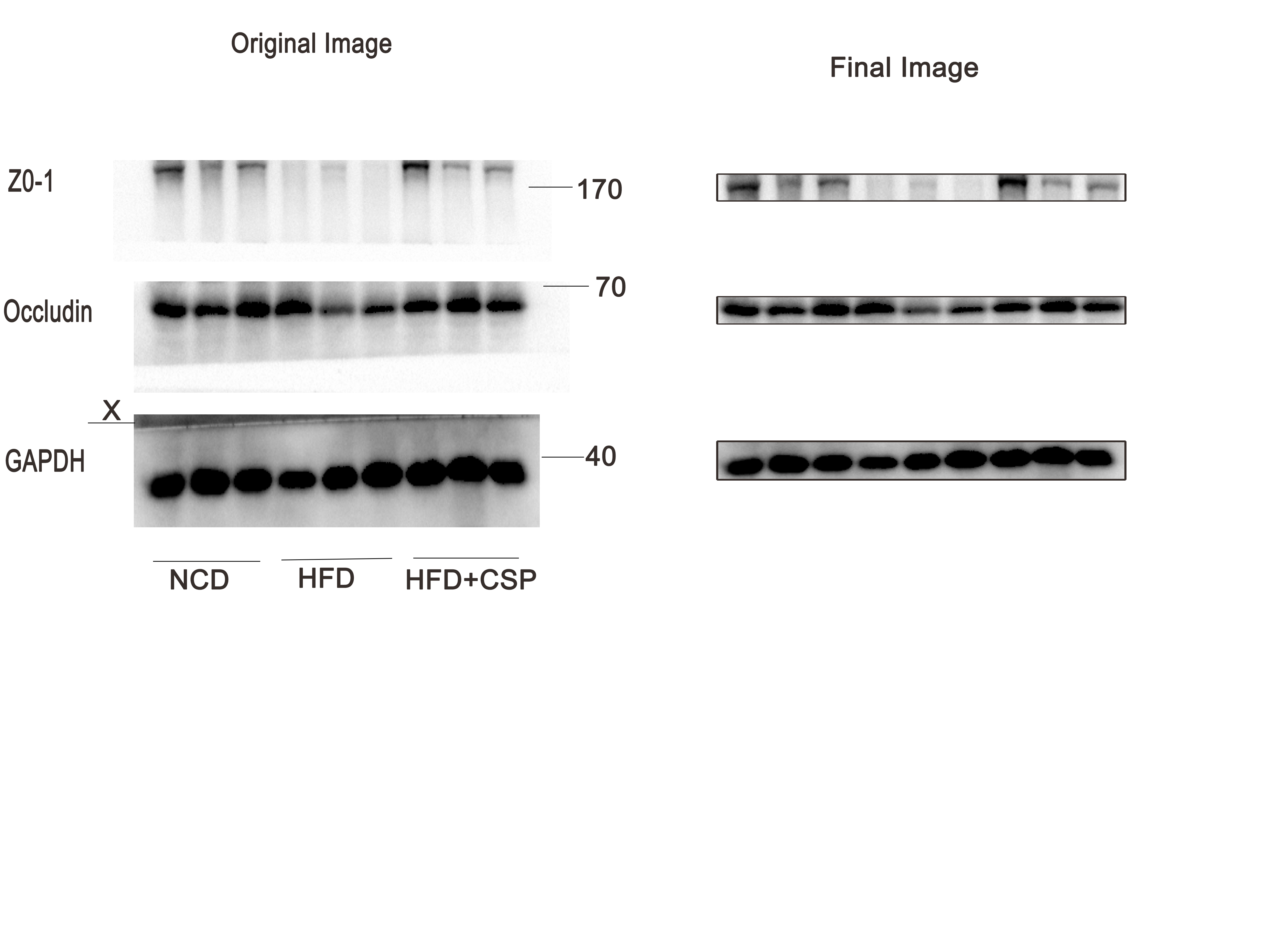

Supplement: S1 Raw images — (TIF) [file pone.0232972.s002.tif]
