# Supplementary material for: Polysaccharides isolated from Cordyceps Sinensis contribute to the progression of NASH by modifying the gut microbiota in mice fed a high-fat diet
Source: PLoS One. 2020 Jun 8;15(6):e0232972. doi: 10.1371/journal.pone.0232972 (PMC7279895; doi:10.1371/journal.pone.0232972)
Supplement: Attachment — Submitted filename: PONE-D-19-33795.edited.docx [file pone.0232972.s003.docx]

**Title:**  **Polysaccharides isolated from *Cordyceps Sinensis* contribute to the progression of NASH by modifying the gut microbiota in mice fed a high-fat diet**

**Authors:** Lei Chen, Liangyu Zhang, Wendong Wang, Wei Qiu, Lei Liu, Anhong Ning, Jing Cao, Min Huang, Mintao Zhong.

Given the limited effectiveness of available non-invasive treatments for obesity, there is a need to develop new therapeutic strategies. The authors of this study assessed the anti-obesity potential of polysaccharides isolated from Cordyceps Sinensis in the animal model (C57BL/6J mice on a high-fat diet). Surprisingly, they found that despite the favorable influence on body mass, adipose tissue amount, and morphology, these polysaccharides had a detrimental effect on the liver leading to NASH.

The authors attempted to explain the pathomechanism responsible for the observed phenomenon. Therefore, they investigated the intestinal barrier function and the composition of intestinal microflora in animals exposed to the tested compounds. They found that treatment with the polysaccharides isolated from Cordyceps Sinensis leads to adverse changes in the composition of the intestinal microflora and increased intestinal barrier permeability, which may be responsible for the development of the liver damage.

The presented work is an example of a complex approach to the research task, and both the research hypothesis and the findings are innovative. The study objectives are clearly defined, and the methodology is appropriately selected to implement that planned research tasks. With this in mind, I found the present paper valuable, and I have only some minor remarks/suggestions.

Minor remarks:

1) General remark: please define the abbreviations occurring in the figures in the figures legends.
2) Introduction: 3^rd^ paragraph, line 12: “The liver is important lipid metabolism,” – the preposition is missing in this sentence.
3) Figure 2, panel E – the quality of the photos could be improved.
4) Figure 3 legend, please change ANOVE into ANOVA.
5) Discussion:

- 1^st^ paragraph, line 7 – shouldn’t the "extract power” be replaced by "extract powder”?

- 3^rd^ paragraph, lines 6-7: "As a result of long-term HFD intake, LDL receptor activity was inhibited in the liver” – please provide a proper reference;

- 3^rd^ paragraph lines 25-32: "The consumption of a HFD will increase the levels of acyl-coenzyme A (acyl-CoA, the activated form of fatty acids), which is an allosteric inhibitor in the cells, and the high concentration of acyl-CoA will inhibit the activity of acetyl CoA carboxylase (ACC), a rate-limiting enzyme that catalyzes the first step of the fatty acid synthesis pathway that produces malonyl-CoA. Palmitic acid biosynthesis is controlled by the *SREBP-1c* and *FAS* genes.” – please provide proper references;

- 4th paragraph lines 17-18: "IR often occurs in type 2 diabetes with weight loss.” – please provide a proper reference;

- 8th paragraph, line 8 – please explain the abbreviation DSS;

- 8th paragraph, lines 8-9: "These findings suggest that purified dietary fiber may have similar side effects on intestinal flora regulation” – please precise the meaning;

- 9th paragraph, line 24 – "In at study on the effect…” – please replace "at" with "a".
