# Supplementary material for: Polysaccharides isolated from Cordyceps Sinensis contribute to the progression of NASH by modifying the gut microbiota in mice fed a high-fat diet
Source: PLoS One. 2020 Jun 8;15(6):e0232972. doi: 10.1371/journal.pone.0232972 (PMC7279895; doi:10.1371/journal.pone.0232972)
Supplement: Attachment — Submitted filename: Response to Reviewers.docx [file pone.0232972.s004.docx]

Part 1(Reviewer #1)

Dear Reviewer:
Thank you for your comments concerning our manuscript entitled “Polysaccharides isolated from Cordyceps Sinensis contribute to the progression of NASH by modifying the gut microbiota in mice fed a high fat diet” (ID: PONE-D-19-33795). Those comments are all valuable and very helpful for revising and improving our paper, as well as the important guiding significance to our researches. We have studied comments carefully and have made Response to your comments. We hope meet with approval.

1. The reviewer’s comment: Can be edited to be briefer.

Response: Thank you for your advice. We considered this comment carefully. Every part of the current manuscript is very important and simplified. If it is shorted, the quality of the article cannot be guaranteed. If you think there are any places that can be modified, please point out and we can make the appropriate edits.

2. The reviewer’s comment: Did the two groups of mice have comparable or identical caloric intake of the chow?

Response: Yes, the two groups of mice have identical caloric intake of the chow. We compared the mice caloric intake of the chow between HFD and HFD+CSP group in 8 weeks, and there is no significant difference between these two groups though ANOVA analysis.

3. The reviewer’s comment: Perhaps some more background in the introduction about why this was chosen to be studied in particular? Is it the most common ingredient in Chinese herbal products?

Response: Thank you for your comment. Cordyceps Sinensis is a kind of expensive Chinese medicine with a very high status in Chinese medicine history. With the advancement of artificial cultivation technology, Cordyceps Sinensis is made into different types of health products or medicines and is marketed with a very large user group. However, manufacturers' sales often will mislead buyers to ignore it was complex ingredients. Besides, there have been reports stating that Chinese medicine and soluble fiber have different degrees of side effects. CSP, as a soluble fiber extracted from traditional Chinese medicine, it is of great value for research. So we support more background to emphasize the importance of why it was chosen to be studied particular.

The Chinese traditional medicine classification includes animals, plants, fungi and others. The CSP was mainly derived from the cell wall of cells, so it is only the most important component in fungal and plant Chinese herbal products. However, polysaccharides extracted from different kinds of Chinese medicines are different.

Special thanks to you for your good comments. We appreciate for your warm work earnestly, and hope that the response will meet with approval.
Once again, thank you very much for your comments and suggestions.

Sincerely yours,

Mintao Zhong.

Part 2(Reviewer #2)

Dear Reviewer:
Thank you for your comments concerning our manuscript entitled “Polysaccharides isolated from Cordyceps Sinensis contribute to the progression of NASH by modifying the gut microbiota in mice fed a high fat diet” (ID: PONE-D-19-33795). Those comments are all valuable and very helpful for revising and improving our paper, as well as the important guiding significance to our researches. We have studied comments carefully and have made correction which we hope meet with approval. Revised portion are marked in red in the paper. The corrections in the paper and the responds to your comments are as flowing:

1. The reviewer’s comment: General remark: please define the abbreviations occurring in the figures in the figures legends.

Response: Thank you for pointing this. In this revised manuscript, we have defined the abbreviation occurring in the figures in the figures legends. Including Fig 1(CSP-I, CSP-II, CSP-I1, CSP-I2, DEAE-52), Fig 2(H&E), Fig 3(CHO, HDL-C, LDL-C, TG, FAS, Srebp1-c, PPARy), Fig 5(LPS, TNF-α, IL-6, MCP-1), Fig 6(ZO-1), Fig 7(OUT, UPGMA, PCA, PCoA),Fig 8 (LEfSe, LDA).

2. The reviewer’s comment: Introduction: 3rd paragraph, line 12: “The liver is important lipid metabolism,” – the preposition is missing in this sentence.

Response: Thank you for pointing that. We have added “in human body” in this sentence.

3. The reviewer’s comment: Figure 2, panel E the quality of the photos could be improved.

Response: We have improved the clarity and brightness in Figure 2, panel E.

4. The reviewer’s comment: Figure 3 legend, please change ANOVE into ANOVA

Response: We are very sorry for our incorrect writing. We have replaced “ANOVE” with “ANOVA” in the revised manuscript.

5. The reviewer’s comment: Discussion - 1st paragraph, line 7 – shouldn’t the "extract power” be replaced by "extract powder”?

Response: We have replaced "extract power” by "extract powder” in the revised manuscript.

6. The reviewer’s comment: Discussion-3rd paragraph, lines 6-7: "As a result of long-term HFD intake, LDL receptor activity was inhibited in the liver” – please provide a proper reference

Response: A proper reference has been added into the revised manuscript.

7. The reviewer’s comment: Discussion-3rd paragraph lines 25-32: "The consumption of a HFD will increase the levels of acyl-coenzyme A (acyl-CoA, the activated form of fatty acids), which is an allosteric inhibitor in the cells, and the high concentration of acyl-CoA will inhibit the activity of acetyl CoA carboxylase (ACC), a rate-limiting enzyme that catalyzes the first step of the fatty acid synthesis pathway that produces malonyl-CoA. Palmitic acid biosynthesis is controlled by the SREBP-1c and FAS genes” – please provide proper references

Response: We have inserted three essential references to explain the lipid metabolism in liver and related genes in the revised manuscript.

8. The reviewer’s comment: Discussion - 4th paragraph lines 17-18: "IR often occurs in type 2 diabetes with weight loss.” -please provide a proper reference;

Response: We have provided a proper reference for the lines in the revised manuscript.

9. The reviewer’s comment: Discussion - 8th paragraph, line 8 – please explain the abbreviation DSS;

Response: Thank you for your advice. We have added “Dextran Sulfate Sodium” to explain the abbreviation DSS in the revised manuscript.

10. The reviewer’s comment: Discussion - 8th paragraph, lines 8-9: "These findings suggest that purified dietary fiber may have similar side effects on intestinal flora regulation” – please precise the meaning

Response: We have added “which means that when the intestinal homeostasis was destroyed, instead of protecting, purified dietary fiber will aggravate the degree of damage, such as inflammation caused by exposure to DSS.” to previse this sentence.

11. The reviewer’ comment: Discussion - 9th paragraph, line 24 – "In at study on the effect…” – please replace "at" with "a".

Response: We have replaced “at” with “a” in the revised manuscript.

Special thanks to you for your good comments. We appreciate for your warm work earnestly, and hope that the correction will meet with approval.
Once again, thank you very much for your comments and suggestions.

Sincerely yours,

Mintao Zhong.
