# Supplementary material for: Polysaccharides isolated from Cordyceps Sinensis contribute to the progression of NASH by modifying the gut microbiota in mice fed a high-fat diet
Source: PLoS One. 2020 Jun 8;15(6):e0232972. doi: 10.1371/journal.pone.0232972 (PMC7279895; doi:10.1371/journal.pone.0232972)
Supplement: Peer review history

# Peer review: Decision Letter 0

**DOI:** 10.1371/journal.pone.0232972.r001

**Authors:** Jonathan M Peterson

12 Mar 2020

PONE-D-19-33795

Polysaccharides isolated from Cordyceps Sinensis contribute to the progression of NASH by modifying the gut microbiota in mice fed a high fat diet

PLOS ONE

Dear Dr. Zhong,

Thank you for submitting your manuscript to PLOS ONE. After careful consideration, we feel that it has merit but does not fully meet PLOS ONE’s publication criteria as it currently stands. Therefore, we invite you to submit a revised version of the manuscript that addresses the points raised during the review process.

There were a small number of minor mostly editorial suggested made by both reviewers that should be considered.

We would appreciate receiving your revised manuscript by Apr 26 2020 11:59PM. When you are ready to submit your revision, log on to https://www.editorialmanager.com/pone/ and select the 'Submissions Needing Revision' folder to locate your manuscript file.

If you would like to make changes to your financial disclosure, please include your updated statement in your cover letter.

To enhance the reproducibility of your results, we recommend that if applicable you deposit your laboratory protocols in protocols.io, where a protocol can be assigned its own identifier (DOI) such that it can be cited independently in the future. For instructions see: http://journals.plos.org/plosone/s/submission-guidelines#loc-laboratory-protocols

Please include the following items when submitting your revised manuscript:

- A rebuttal letter that responds to each point raised by the academic editor and reviewer(s). This letter should be uploaded as separate file and labeled 'Response to Reviewers'.
- A marked-up copy of your manuscript that highlights changes made to the original version. This file should be uploaded as separate file and labeled 'Revised Manuscript with Track Changes'.
- An unmarked version of your revised paper without tracked changes. This file should be uploaded as separate file and labeled 'Manuscript'.

Please note while forming your response, if your article is accepted, you may have the opportunity to make the peer review history publicly available. The record will include editor decision letters (with reviews) and your responses to reviewer comments. If eligible, we will contact you to opt in or out.

We look forward to receiving your revised manuscript.

Kind regards,

Jonathan M Peterson, Ph.D.

Academic Editor

PLOS ONE

Additional Editor Comments (if provided):

A small number of minor revisions to the test are requested by both reviewers.

Journal Requirements:

When submitting your revision, we need you to address these additional requirements:

1. Please ensure that your manuscript meets PLOS ONE's style requirements, including those for file naming. The PLOS ONE style templates can be found at http://www.plosone.org/attachments/PLOSOne_formatting_sample_main_body.pdf and http://www.plosone.org/attachments/PLOSOne_formatting_sample_title_authors_affiliations.pdf

2. We note that you are reporting an analysis of a microarray, next-generation sequencing, or deep sequencing data set. PLOS requires that authors comply with field-specific standards for preparation, recording, and deposition of data in repositories appropriate to their field. Please upload these data to a stable, public repository (such as ArrayExpress, Gene Expression Omnibus (GEO), DNA Data Bank of Japan (DDBJ), NCBI GenBank, NCBI Sequence Read Archive, or EMBL Nucleotide Sequence Database (ENA)). In your revised cover letter, please provide the relevant accession numbers that may be used to access these data. For a full list of recommended repositories, see http://journals.plos.org/plosone/s/data-availability#loc-omics or http://journals.plos.org/plosone/s/data-availability#loc-sequencing.

3. PLOS ONE now requires that authors provide the original uncropped and unadjusted images underlying all blot or gel results reported in a submission’s figures or Supporting Information files. This policy and the journal’s other requirements for blot/gel reporting and figure preparation are described in detail at https://journals.plos.org/plosone/s/figures#loc-blot-and-gel-reporting-requirements and https://journals.plos.org/plosone/s/figures#loc-preparing-figures-from-image-files. When you submit your revised manuscript, please ensure that your figures adhere fully to these guidelines and provide the original underlying images for all blot or gel data reported in your submission. See the following link for instructions on providing the original image data: https://journals.plos.org/plosone/s/figures#loc-original-images-for-blots-and-gels.

In your cover letter, please note whether your blot/gel image data are in Supporting Information or posted at a public data repository, provide the repository URL if relevant, and provide specific details as to which raw blot/gel images, if any, are not available. Email us at plosone@plos.org if you have any questions.

4. PLOS requires an ORCID iD for the corresponding author in Editorial Manager on papers submitted after December 6th, 2016. Please ensure that you have an ORCID iD and that it is validated in Editorial Manager. To do this, go to ‘Update my Information’ (in the upper left-hand corner of the main menu), and click on the Fetch/Validate link next to the ORCID field. This will take you to the ORCID site and allow you to create a new iD or authenticate a pre-existing iD in Editorial Manager. Please see the following video for instructions on linking an ORCID iD to your Editorial Manager account: https://www.youtube.com/watch?v=_xcclfuvtxQ

5. Your ethics statement must appear in the Methods section of your manuscript. If your ethics statement is written in any section besides the Methods, please move it to the Methods section and delete it from any other section. Please also ensure that your ethics statement is included in your manuscript, as the ethics section of your online submission will not be published alongside your manuscript.

6. Please include captions for your Supporting Information files at the end of your manuscript, and update any in-text citations to match accordingly. Please see our Supporting Information guidelines for more information: http://journals.plos.org/plosone/s/supporting-information.

[Note: HTML markup is below. Please do not edit.]

Reviewers' comments:

Reviewer's Responses to Questions

**Comments to the Author**

1. Is the manuscript technically sound, and do the data support the conclusions?

The manuscript must describe a technically sound piece of scientific research with data that supports the conclusions. Experiments must have been conducted rigorously, with appropriate controls, replication, and sample sizes. The conclusions must be drawn appropriately based on the data presented. 

Reviewer #1: Yes

Reviewer #2: Yes

**********

2. Has the statistical analysis been performed appropriately and rigorously? 

Reviewer #1: Yes

Reviewer #2: Yes

**********

3. Have the authors made all data underlying the findings in their manuscript fully available?

The PLOS Data policy requires authors to make all data underlying the findings described in their manuscript fully available without restriction, with rare exception (please refer to the Data Availability Statement in the manuscript PDF file). The data should be provided as part of the manuscript or its supporting information, or deposited to a public repository. For example, in addition to summary statistics, the data points behind means, medians and variance measures should be available. If there are restrictions on publicly sharing data—e.g. participant privacy or use of data from a third party—those must be specified.

Reviewer #1: Yes

Reviewer #2: Yes

**********

4. Is the manuscript presented in an intelligible fashion and written in standard English?

PLOS ONE does not copyedit accepted manuscripts, so the language in submitted articles must be clear, correct, and unambiguous. Any typographical or grammatical errors should be corrected at revision, so please note any specific errors here.

Reviewer #1: Yes

Reviewer #2: Yes

**********

5. Review Comments to the Author

Please use the space provided to explain your answers to the questions above. You may also include additional comments for the author, including concerns about dual publication, research ethics, or publication ethics. (Please upload your review as an attachment if it exceeds 20,000 characters)

Reviewer #1: 1. Can be edited to be briefer

2. Did the two groups of mice have comparable or identical caloric intake of the chow?

3. Perhaps some more background in the introduction about why this was chosen to be studied in particular? Is it the most common ingredient in Chinese herbal products?

Reviewer #2: Given the limited effectiveness of available non-invasive treatments for obesity, there is a need to develop new therapeutic strategies. The authors of this study assessed the anti-obesity potential of polysaccharides isolated from Cordyceps Sinensis in the animal model (C57BL/6J mice on a high-fat diet). Surprisingly, they found that despite the favorable influence on body mass, adipose tissue amount, and morphology, these polysaccharides had a detrimental effect on the liver leading to NASH.

The authors attempted to explain the pathomechanism responsible for the observed phenomenon. Therefore, they investigated the intestinal barrier function and the composition of intestinal microflora in animals exposed to the tested compounds. They found that treatment with the polysaccharides isolated from Cordyceps Sinensis leads to adverse changes in the composition of the intestinal microflora and increased intestinal barrier permeability, which may be responsible for the development of the liver damage.

The presented work is an example of a complex approach to the research task, and both the research hypothesis and the findings are innovative. The study objectives are clearly defined, and the methodology is appropriately selected to implement that planned research tasks. With this in mind, I found the present paper valuable, and I have only some minor remarks/suggestions that are summarized in the attachment.

**********

6. PLOS authors have the option to publish the peer review history of their article (what does this mean?). If published, this will include your full peer review and any attached files.

If you choose “no”, your identity will remain anonymous but your review may still be made public.

**Do you want your identity to be public for this peer review?** For information about this choice, including consent withdrawal, please see our Privacy Policy.

Reviewer #1: Yes: Vinod Kumar Rustgi

Reviewer #2: No

[NOTE: If reviewer comments were submitted as an attachment file, they will be attached to this email and accessible via the submission site. Please log into your account, locate the manuscript record, and check for the action link "View Attachments". If this link does not appear, there are no attachment files to be viewed.]

While revising your submission, please upload your figure files to the Preflight Analysis and Conversion Engine (PACE) digital diagnostic tool, https://pacev2.apexcovantage.com/. PACE helps ensure that figures meet PLOS requirements. To use PACE, you must first register as a user. Registration is free. Then, login and navigate to the UPLOAD tab, where you will find detailed instructions on how to use the tool. If you encounter any issues or have any questions when using PACE, please email us at figures@plos.org. Please note that Supporting Information files do not need this step.

Attachment Submitted filename: PONE-D-19-33795.edited.docx Click here for additional data file.

# Author comment: Author response to Decision Letter 0

**DOI:** 10.1371/journal.pone.0232972.r002

16 Apr 2020

Part 1(Reviewer #1)

Dear Reviewer:

Thank you for your comments concerning our manuscript entitled “Polysaccharides isolated from Cordyceps Sinensis contribute to the progression of NASH by modifying the gut microbiota in mice fed a high fat diet” (ID: PONE-D-19-33795). Those comments are all valuable and very helpful for revising and improving our paper, as well as the important guiding significance to our researches. We have studied comments carefully and have made Response to your comments. We hope meet with approval. 

1. The reviewer’s comment: Can be edited to be briefer.

Response: Thank you for your advice. We considered this comment carefully. Every part of the current manuscript is very important and simplified. If it is shorted, the quality of the article cannot be guaranteed. If you think there are any places that can be modified, please point out and we can make the appropriate edits. 

2. The reviewer’s comment: Did the two groups of mice have comparable or identical caloric intake of the chow?

Response: Yes, the two groups of mice have identical caloric intake of the chow. We compared the mice caloric intake of the chow between HFD and HFD+CSP group in 8 weeks, and there is no significant difference between these two groups though ANOVA analysis.

3. The reviewer’s comment: Perhaps some more background in the introduction about why this was chosen to be studied in particular? Is it the most common ingredient in Chinese herbal products?

Response: Thank you for your comment. Cordyceps Sinensis is a kind of expensive Chinese medicine with a very high status in Chinese medicine history. With the advancement of artificial cultivation technology, Cordyceps Sinensis is made into different types of health products or medicines and is marketed with a very large user group. However, manufacturers' sales often will mislead buyers to ignore it was complex ingredients. Besides, there have been reports stating that Chinese medicine and soluble fiber have different degrees of side effects. CSP, as a soluble fiber extracted from traditional Chinese medicine, it is of great value for research. So we support more background to emphasize the importance of why it was chosen to be studied particular.

The Chinese traditional medicine classification includes animals, plants, fungi and others. The CSP was mainly derived from the cell wall of cells, so it is only the most important component in fungal and plant Chinese herbal products. However, polysaccharides extracted from different kinds of Chinese medicines are different.

Special thanks to you for your good comments. We appreciate for your warm work earnestly, and hope that the response will meet with approval.

Once again, thank you very much for your comments and suggestions.

Sincerely yours,

Mintao Zhong.

Part 2(Reviewer #2)

Dear Reviewer:

Thank you for your comments concerning our manuscript entitled “Polysaccharides isolated from Cordyceps Sinensis contribute to the progression of NASH by modifying the gut microbiota in mice fed a high fat diet” (ID: PONE-D-19-33795). Those comments are all valuable and very helpful for revising and improving our paper, as well as the important guiding significance to our researches. We have studied comments carefully and have made correction which we hope meet with approval. Revised portion are marked in red in the paper. The corrections in the paper and the responds to your comments are as flowing:

1. The reviewer’s comment: General remark: please define the abbreviations occurring in the figures in the figures legends.

Response: Thank you for pointing this. In this revised manuscript, we have defined the abbreviation occurring in the figures in the figures legends. Including Fig 1(CSP-I, CSP-II, CSP-I1, CSP-I2, DEAE-52), Fig 2(H&E), Fig 3(CHO, HDL-C, LDL-C, TG, FAS, Srebp1-c, PPARy), Fig 5(LPS, TNF-α, IL-6, MCP-1), Fig 6(ZO-1), Fig 7(OUT, UPGMA, PCA, PCoA),Fig 8 (LEfSe, LDA).

2. The reviewer’s comment: Introduction: 3rd paragraph, line 12: “The liver is important lipid metabolism,” – the preposition is missing in this sentence.

Response: Thank you for pointing that. We have added “in human body” in this sentence.

3. The reviewer’s comment: Figure 2, panel E the quality of the photos could be improved.

Response: We have improved the clarity and brightness in Figure 2, panel E.

4. The reviewer’s comment: Figure 3 legend, please change ANOVE into ANOVA

Response: We are very sorry for our incorrect writing. We have replaced “ANOVE” with “ANOVA” in the revised manuscript.

5. The reviewer’s comment: Discussion - 1st paragraph, line 7 – shouldn’t the "extract power” be replaced by "extract powder”?

Response: We have replaced "extract power” by "extract powder” in the revised manuscript. 

6. The reviewer’s comment: Discussion-3rd paragraph, lines 6-7: "As a result of long-term HFD intake, LDL receptor activity was inhibited in the liver” – please provide a proper reference

Response: A proper reference has been added into the revised manuscript. 

7. The reviewer’s comment: Discussion-3rd paragraph lines 25-32: "The consumption of a HFD will increase the levels of acyl-coenzyme A (acyl-CoA, the activated form of fatty acids), which is an allosteric inhibitor in the cells, and the high concentration of acyl-CoA will inhibit the activity of acetyl CoA carboxylase (ACC), a rate-limiting enzyme that catalyzes the first step of the fatty acid synthesis pathway that produces malonyl-CoA. Palmitic acid biosynthesis is controlled by the SREBP-1c and FAS genes” – please provide proper references

Response: We have inserted three essential references to explain the lipid metabolism in liver and related genes in the revised manuscript. 

8. The reviewer’s comment: Discussion - 4th paragraph lines 17-18: "IR often occurs in type 2 diabetes with weight loss.” -please provide a proper reference;

Response: We have provided a proper reference for the lines in the revised manuscript. 

9. The reviewer’s comment: Discussion - 8th paragraph, line 8 – please explain the abbreviation DSS;

Response: Thank you for your advice. We have added “Dextran Sulfate Sodium” to explain the abbreviation DSS in the revised manuscript.

10. The reviewer’s comment: Discussion - 8th paragraph, lines 8-9: "These findings suggest that purified dietary fiber may have similar side effects on intestinal flora regulation” – please precise the meaning

Response: We have added “which means that when the intestinal homeostasis was destroyed, instead of protecting, purified dietary fiber will aggravate the degree of damage, such as inflammation caused by exposure to DSS.” to previse this sentence.

11. The reviewer’ comment: Discussion - 9th paragraph, line 24 – "In at study on the effect…” – please replace "at" with "a".

Response: We have replaced “at” with “a” in the revised manuscript.

Special thanks to you for your good comments. We appreciate for your warm work earnestly, and hope that the correction will meet with approval.

Once again, thank you very much for your comments and suggestions.

Sincerely yours,

Mintao Zhong.

Attachment Submitted filename: Response to Reviewers.docx Click here for additional data file.

# Peer review: Decision Letter 1

**DOI:** 10.1371/journal.pone.0232972.r003

**Authors:** Jonathan M Peterson

27 Apr 2020

Polysaccharides isolated from Cordyceps Sinensis contribute to the progression of NASH by modifying the gut microbiota in mice fed a high fat diet

PONE-D-19-33795R1

Dear Dr. Zhong,

We are pleased to inform you that your manuscript has been judged scientifically suitable for publication and will be formally accepted for publication once it complies with all outstanding technical requirements.

Within one week, you will receive an e-mail containing information on the amendments required prior to publication. When all required modifications have been addressed, you will receive a formal acceptance letter and your manuscript will proceed to our production department and be scheduled for publication.

Shortly after the formal acceptance letter is sent, an invoice for payment will follow. To ensure an efficient production and billing process, please log into Editorial Manager at https://www.editorialmanager.com/pone/, click the "Update My Information" link at the top of the page, and update your user information. If you have any billing related questions, please contact our Author Billing department directly at authorbilling@plos.org.

If your institution or institutions have a press office, please notify them about your upcoming paper to enable them to help maximize its impact. If they will be preparing press materials for this manuscript, you must inform our press team as soon as possible and no later than 48 hours after receiving the formal acceptance. Your manuscript will remain under strict press embargo until 2 pm Eastern Time on the date of publication. For more information, please contact onepress@plos.org.

With kind regards,

Jonathan M Peterson, Ph.D.

Academic Editor

PLOS ONE

Additional Editor Comments (optional):

All request revisions were completed and reviewer comments were addressed accordingly.

Reviewers' comments:

Reviewer's Responses to Questions

**Comments to the Author**

1. If the authors have adequately addressed your comments raised in a previous round of review and you feel that this manuscript is now acceptable for publication, you may indicate that here to bypass the “Comments to the Author” section, enter your conflict of interest statement in the “Confidential to Editor” section, and submit your "Accept" recommendation.

Reviewer #2: All comments have been addressed

**********

2. Is the manuscript technically sound, and do the data support the conclusions?

The manuscript must describe a technically sound piece of scientific research with data that supports the conclusions. Experiments must have been conducted rigorously, with appropriate controls, replication, and sample sizes. The conclusions must be drawn appropriately based on the data presented. 

Reviewer #2: Yes

**********

3. Has the statistical analysis been performed appropriately and rigorously? 

Reviewer #2: Yes

**********

4. Have the authors made all data underlying the findings in their manuscript fully available?

The PLOS Data policy requires authors to make all data underlying the findings described in their manuscript fully available without restriction, with rare exception (please refer to the Data Availability Statement in the manuscript PDF file). The data should be provided as part of the manuscript or its supporting information, or deposited to a public repository. For example, in addition to summary statistics, the data points behind means, medians and variance measures should be available. If there are restrictions on publicly sharing data—e.g. participant privacy or use of data from a third party—those must be specified.

Reviewer #2: Yes

**********

5. Is the manuscript presented in an intelligible fashion and written in standard English?

PLOS ONE does not copyedit accepted manuscripts, so the language in submitted articles must be clear, correct, and unambiguous. Any typographical or grammatical errors should be corrected at revision, so please note any specific errors here.

Reviewer #2: Yes

**********

6. Review Comments to the Author

Please use the space provided to explain your answers to the questions above. You may also include additional comments for the author, including concerns about dual publication, research ethics, or publication ethics. (Please upload your review as an attachment if it exceeds 20,000 characters)

Reviewer #2: The authors have significantly improved the manuscript and clarified my doubts. Therefore, I find the paper suitable for publication in a present form.

**********

7. PLOS authors have the option to publish the peer review history of their article (what does this mean?). If published, this will include your full peer review and any attached files.

If you choose “no”, your identity will remain anonymous but your review may still be made public.

**Do you want your identity to be public for this peer review?** For information about this choice, including consent withdrawal, please see our Privacy Policy.

Reviewer #2: Yes: Alina Kurylowicz

# Editor report: Acceptance letter

**DOI:** 10.1371/journal.pone.0232972.r004

**Authors:** Jonathan M Peterson

13 May 2020

PONE-D-19-33795R1 

Polysaccharides isolated from Cordyceps Sinensis contribute to the progression of NASH by modifying the gut microbiota in mice fed a high-fat diet 

Dear Dr. Zhong:

I am pleased to inform you that your manuscript has been deemed suitable for publication in PLOS ONE. Congratulations! Your manuscript is now with our production department. 

If your institution or institutions have a press office, please notify them about your upcoming paper at this point, to enable them to help maximize its impact. If they will be preparing press materials for this manuscript, please inform our press team within the next 48 hours. Your manuscript will remain under strict press embargo until 2 pm Eastern Time on the date of publication. For more information please contact onepress@plos.org.

For any other questions or concerns, please email plosone@plos.org. 

Thank you for submitting your work to PLOS ONE.

With kind regards,

PLOS ONE Editorial Office Staff

on behalf of

Dr Jonathan M Peterson 

Academic Editor

PLOS ONE
